# Demographic characteristics and psychosocial backgrounds of patients with dextromethorphan overdose in Japan: A multicenter prospective observational study

**DOI:** 10.1002/pcn5.70406

**Published:** 2026-09-03

**Authors:** Hironori Katsuki, Yoshito Kamijo, Michiko Takai, Saeko Kohara, Hidetada Fukushima, Yuta Mizuno, Hidenobu Ochiai, Norio Otani, Dai Taguchi, Takuya Shimane, Ryoko Kyan

**Affiliations:** ^1^ Department of Clinical Toxicology Saitama Medical University Hospital Iruma‐gun Saitama Japan; ^2^ Department of Emergency Medicine Iizuka Hospital Iizukashi Fukuoka Japan; ^3^ Department of Critical Care Medicine and Trauma National Hospital Organization Disaster Medical Center Tokyo Tachikawa Japan; ^4^ Department of Emergency and Critical Care Medicine Nara Medical University Kashihara Nara Japan; ^5^ Department of Emergency and Critical Care Medicine Maebashi Red Cross Hospital Maebashi Gunma Japan; ^6^ Department of Emergency, Critical Care, and Disaster Medicine University of Miyazaki Hospital Miyazaki Miyazaki Japan; ^7^ Department of Emergency and Critical Care Medicine St. Luke's International Hospital Tokyo Japan; ^8^ Department of Emergency Medicine Kin‐ikyo Chuo Hospital Sapporo Japan; ^9^ Department of Drug Dependence Research National Institute of Mental Health, National Center of Neurology and Psychiatry Kodaira Japan

**Keywords:** adolescent, dextromethorphan, drug overdose, internet, young adult

## Abstract

**Aim:**

Few studies in Japan have described young patients presenting to emergency departments after dextromethorphan overdose in detail. Therefore, we aimed to investigate the demographic characteristics and psychosocial backgrounds of patients who presented to emergency departments in Japan after dextromethorphan overdose.

**Methods:**

This was a multicenter, prospective, observational study with nonconsecutive enrollment. A standardized questionnaire regarding dextromethorphan overdose and the Drug Abuse Screening Test‐20 (DAST‐20) were administered in eight emergency departments in Japan. Continuous variables are presented as medians and interquartile ranges.

**Results:**

In total, 78 patients were included. The median age was 20 years (17.0–22.0), and 64 patients (82.1%) were female. Fifty‐six (71.8%) patients engaged in social activities. Fifty‐one patients (65.4%) stated that the purpose of the overdose was suicide/self‐harm. The number of patients who reported that the source of information on dextromethorphan overdose was social networking services and internet searches was 27 (35.1%) and 29 (37.7%), respectively. Fifty‐eight patients (74.4%) reported that dextromethorphan was purchased from a retail store. Among 66 respondents, 40 (60.6%) had moderate or higher DAST‐20 scores. On comparing the younger group aged 10–19 years (*n* = 38) and the older group aged ≥20 years (*n* = 40), the number of patients who indicated that the source of information on dextromethorphan overdose was internet searches in the former group (19, 50.0%) was significantly higher than that in the latter group (10, 25.0%).

**Conclusion:**

Dextromethorphan overdose predominantly involved young women, and drug‐related problems were common.

## INTRODUCTION

Suicide has become a major social issue among young people in Japan. The total number of suicides in Japan has shown an overall downward trend; the total number of suicide deaths in 2024 was 20,320, representing a decrease of 1517 from 2023.^1^ However, when examined by age and gender, while the number of suicides decreased from the previous year in most age and gender categories, the number of female suicides increased by 51 from 2023, accounting for 430 of the 800 suicides among individuals aged 19 years or younger.^1^ Therefore, strengthening suicide prevention measures among young people is an urgent priority in Japan.

Among the various methods of suicide attempts used by young people, overdoses on over‐the‐counter (OTC) medications have been increasingly reported in Japan.^2^, ^3^ One factor underlying this trend is the growing problem of dependence on and misuse of OTC medications among adolescents and young adults. A nationwide survey of psychiatric treatment facilities in Japan showed that among patients receiving treatment for drug dependence, the proportion of adolescent patients whose primary drug was an OTC medication increased from 25.0% in 2016 to 56.4% in 2020, 65.2% in 2022, and 71.5% in 2024.^4^ In addition, in a secondary analysis of data from the Nationwide High School Survey on Drug Use and Lifestyle 2021, Shimane et al. analyzed the responses to self‐administered questionnaires from 41,357 participants and showed that the prevalence of OTC medication misuse was 1.5%.^5^ In a database study using data from the National Psychiatric Hospital Survey (NPH Survey), Usami et al. reported that the peak age of individuals misusing OTC medications shifted from 30s to 40s in 2018 before the coronavirus disease 2019 (COVID‐19) pandemic to 20s to 30s in 2022 after the pandemic.^6^ In response to the growing recognition that dependence on and misuse of OTC medications have spread among young people, Japan began regulating the following six substances in 2022 as part of its measures against dependence on and misuse of non‐prescription drugs: ephedrine, codeine, dihydrocodeine, bromovalerylurea, pseudoephedrine, and methylephedrine.^7^

Recent detailed examinations of the patterns of overdose involving OTC medications in Japan have revealed that overdose on dextromethorphan, which had not been previously subject to regulation, has become increasingly prevalent.^2^, ^3^, ^6^ Therefore, dextromethorphan may be an important substance used by young people who overdose on OTC medications. However, to our knowledge, no study in Japan has described young patients who presented to emergency departments (EDs) after dextromethorphan overdose in detail. Therefore, in this multicenter study, we aimed to describe the demographic characteristics and psychosocial backgrounds of patients who presented to EDs in Japan after dextromethorphan overdose, with a particular focus on younger patients, and to compare these characteristics between patients aged 10–19 years and those aged 20 years or older.

## METHODS

### Participants

This prospective multicenter observational study was conducted in eight EDs in Japan, including those in university and general hospitals providing both secondary‐ and tertiary‐level emergency care. The participants were patients who presented to the ED with symptoms or signs of acute poisoning between July 10, 2023, and December 31, 2025, and from whom consent to study participation was obtained. Patients were included if the treating physician judged that they had overdosed on dextromethorphan based on self‐report, information obtained from family members or other relevant persons, and/or supporting evidence, such as empty medication packages. Eligible patients were enrolled nonconsecutively. Screening logs were not maintained. No additional exclusion criteria were applied. For minor patients, an explanation was provided using an assent document, and consent was obtained from both the patient and their parent or legal guardian. This study was designed to describe the demographic and psychosocial backgrounds of patients presenting with dextromethorphan overdose. Therefore, no primary or secondary outcomes were predefined.

### Ethics approval statement

This study was performed with approval from the Institutional Review Board of the Ethics Committee of Saitama Medical University Hospital (2023‐027).

### Procedures

To reduce information bias, data were collected using a standardized questionnaire that was shared across the participating sites. The questionnaire was sent by e‐mail to multiple participating EDs. The treating physician completed the questionnaire when the patient was discharged from the hospital or ED.

In addition, patients were asked to complete the Drug Abuse Screening Test‐20 (DAST‐20).^8^

Finally, the questionnaire and DAST‐20 were mailed to the Clinical Toxicology Center of Saitama Medical University Hospital.

### Measures

The questionnaire included items to collect the following information: age, sex, occupation, marital status, presence or absence of cohabitants, medical history, source of dextromethorphan acquisition, source of information on dextromethorphan overdose, history of previous dextromethorphan overdose, purpose of overdose, length of hospital stay in a general medical ward, clinical outcomes, and follow‐up after discharge. Based on their occupation, patients were classified into seven categories: full‐time employment, part‐time employment/casual employment, unemployed, student, homemaker, leave of absence from school/work, and unknown. For the purposes of this study, engagement in social activities was operationally defined according to occupational status as participation in a regular societal role, including full‐time employment, part‐time or casual employment, student status, or homemaking. This was a study‐specific classification and was not based on a validated measure of social functioning or social participation. Regarding overdose, multiple answers were allowed for the following items: seeking the intended effect, recreation/euphoria, relaxation, sleep, inability to stop, suicide/self‐harm, escape from reality, wanting to feel better, none in particular, and others. To determine the source of dextromethorphan acquisition, multiple answers were allowed for the following categories: purchase from a retail store, medication owned by a family member, medication owned by a friend, internet purchase, and others. Regarding the source of information on dextromethorphan overdose, multiple answers were allowed for the following categories: social networking services (SNSs), internet searches, retail stores, friends, and others. The clinical outcomes were classified into three categories: death, residual sequelae, and full recovery. Residual sequelae and full recovery were determined by the treating physician based on the overall clinical assessment at the time of discharge because no pre‐specified objective criteria were used.

The Japanese version of the DAST‐20—a self‐administered assessment scale for drug dependence—was used.^9^ The DAST‐20 scores were categorized into four severity levels: mild (1–5), moderate (6–10), severe (11–15), and very severe (16–20).

### Data analyses

Because this was a descriptive observational study, no formal sample size calculations were performed. All eligible patients who provided consent during the study period were included. The demographic characteristics, psychosocial backgrounds, clinical outcomes, and DAST‐20 scores of the included patients were evaluated. Continuous variables were described as medians and interquartile ranges (IQRs), and nominal or binary variables were described as numbers and percentages. The age cutoff was set at 20 years because the Japanese national suicide statistics commonly distinguish individuals aged 19 years or younger from those in their twenties. Accordingly, patients were divided into a younger group (10–19 years) and an older group (20 years or older). For the between‐group comparison, the DAST‐20 severity categories were dichotomized as mild (1–5) and moderate or greater (6–20). Continuous variables were compared using the Mann–Whitney *U* test, and binary and nominal variables were compared using Fisher's exact test. Statistical analyses were performed using EZR version 1.68 (Saitama Medical Center, Jichi Medical University, Saitama, Japan),^10^ and the significance level was set at 0.05. Missing data were not imputed. Each analysis was conducted using available data for the variables of interest.

## RESULTS

### Demographic characteristics

As consecutive screening was not performed, the number of patients who were potentially eligible, assessed for eligibility, or were not enrolled was unavailable. A total of 78 patients were included in this study. Analyses were performed using available data, and the denominator for each variable was shown if data were missing. The demographic characteristics of the participants are shown in Table 1. The median age of the patients was 20.0 years (IQR, 17.0–22.0 years), and 38 (48.7%) patients were aged 10–19 years. Sixty‐four (82.1%) patients were female. Sixty‐two (79.5%) patients were never married, and 63 (80.8%) had cohabitants. Although 56 (71.8%) patients were engaged in some form of social activity, 16 (20.5%) patients were unemployed, and 6 (7.7%) patients were on leave of absence from work or school.

**Table 1:** Demographic characteristics of patients with dextromethorphan overdose in Japan.

Variables	Overall (*n* = 78)	Missing, *n*
Age years, median [IQR]	20.0 [17.0–22.0]	0
Age category, *n* (%)		
10–19 years	38 (48.7)	
20–29 years	31 (39.7)	
30–39 years	7 (9.0)	
≥40 years	2 (2.6)	
Female, *n* (%)	64 (82.1)	0
Occupation, *n* (%)		0
Full‐time employment	10 (12.8)	
Part‐time employment/casual employment	13 (16.7)	
Unemployed	16 (20.5)	
Student	30 (38.5)	
Homemaker	0 (0.0)	
Leave of absence from school/work	6 (7.7)	
Unknown	3 (3.8)	
Social activities, *n* (%)	53 (67.9)	
Marital status, *n* (%)		0
Married	3 (3.8)	
Never married	62 (79.5)	
Divorced	12 (15.4)	
Widowed	1 (1.3)	
Common‐law relationship	0 (0.0)	
Cohabitants, *n* (%)		0
Yes	63 (80.8)	
No	14 (17.9)	
Unknown	1 (1.3)	

*Note*: These numbers may not add up to the total because of missing data.

Abbreviation: IQR, interquartile range.

### Psychosocial backgrounds

Table 2 summarizes the psychosocial backgrounds related to overdose in the study population. The most common source of dextromethorphan was retail stores (58 [74.4%] patients). The most common source of information on dextromethorphan overdose was internet searches (29 [37.7%] patients), followed by SNS (27 [35.1%] patients). Only 12 (15.6%) patients reported obtaining this information from acquaintances or friends.

**Table 2:** Psychosocial backgrounds related to dextromethorphan overdose in Japan.

Variable	Overall (*n* = 78)	Missing, *n*
History of physical illness, *n* (%)		0
Yes	14 (17.9)	
No	60 (76.9)	
Unknown	4 (5.1)	
History of psychiatric disorders, *n* (%)		0
Yes	57 (73.1)	
No	20 (25.6)	
Unknown	1 (1.3)	
First dextromethorphan overdose, *n* (%)	14 (17.9)	3
Source of dextromethorphan acquisition (total 81 cases), *n* (%)		
Purchase from a retail store	58 (74.4)	
Medication owned by a family member	3 (3.9)	
Medication owned by a friend	5 (6.4)	
Internet purchase	12 (15.4)	
Others	3 (3.8)	
Source of information on dextromethorphan overdose (total 87 cases), *n* (%)		
SNS	27 (35.1)	
Internet searches	29 (37.7)	
Retail stores	12 (15.6)	
Friends	12 (15.6)	
Others	7 (9.1)	
Purpose of overdose (total 122 cases), *n* (%)		
Suicide/self‐harm	51 (65.4)	
Escape from reality	27 (34.6)	
Unable to stop	11 (14.1)	
Wanting to feel better	6 (7.7)	
Recreation/euphoria	6 (7.7)	
Relaxation	4 (5.1)	
Sleep	2 (2.6)	
Seeking the intended effect	0 (0.0)	
None in particular	3 (3.9)	
Others	12 (15.4)	

*Note*: Multiple responses were allowed for the purpose of the overdose, source of dextromethorphan acquisition, and source of information on dextromethorphan overdose.

Abbreviation: SNS, social networking service.

The most frequently reported purpose of overdose was suicide/self‐harm (51 [65.4%] patients), followed by escape from reality (27 [34.6%] patients) and inability to stop (11 [14.1%] patients). By contrast, only 6 (7.7%) patients reported recreation/euphoria.

### Clinical outcomes and DAST‐20 scores

The clinical outcomes and DAST‐20 scores of the study population are shown in Table 3. The median length of stay in the general medical ward was 2.0 days (IQR, 1.0–3.0). None of the patients died, and almost all achieved full recovery (74 [98.7%] patients). More than 60% of the patients were followed up by a psychiatrist after discharge. The median DAST‐20 score after admission was 7.0 (IQR, 5.0–9.0). The severity level was mild in 28 (39.4%) patients, whereas it was moderate or greater in 43 (60.6%) patients.

**Table 3:** Clinical outcomes and Drug Abuse Screening Test‐20 (DAST‐20) results in patients with dextromethorphan overdose in Japan.

Variable	Overall (*n* = 78)	Missing, *n*
Length of hospital stay, median [IQR]	2.0 [1.0–3.0]	2
Clinical outcome, *n* (%)		2
Death	0 (0.0)	
Residual sequelae	1 (1.3)	
Full recovery	74 (98.7)	
Follow‐up after discharge, *n* (%)		1
None	15 (19.5)	
Psychiatric department	53 (68.8)	
Non‐psychiatric department	3 (3.9)	
Both psychiatric and non‐psychiatric departments	6 (7.8)	
DAST‐20, median [IQR]	7.0 [5.0ｰ9.0]	7
DAST‐20 category, *n* (%)		
Mild	28 (39.4)	
Moderate	34 (47.9)	
Severe	9 (12.7)	
Very severe	0 (0.0)	

*Note*: These numbers may not add up to the total because of missing data. The DAST‐20 scores were only available for patients who completed the questionnaires.

Abbreviation: IQR, interquartile range.

### Comparison between the younger and older groups

A comparison between the younger (38 [48.7%] patients) and older (40 [51.3%] patients) groups is shown in Table 4. The proportion of patients who had cohabitants was larger in the younger group than in the older group (35 patients [92.1%] vs. 28 [70.0%], *p* = 0.007). Additionally, internet searches were significantly more frequently reported as a source of information on dextromethorphan overdose in the younger group (19 [50.0%] vs. 10 [25.0%] patients, *p* = 0.02).

**Table 4:** Comparison of demographic characteristics, psychosocial backgrounds, clinical outcomes, and Drug Abuse Screening Test‐20 (DAST‐20) results between the younger and older groups of patients with dextromethorphan overdose.

Variables	Younger, *n* = 38	Older, *n* = 40	*p* value
Female, *n* (%)	31 (81.6)	33 (82.5)	1
Social activities, *n* (%)	28 (73.7)	25 (62.5)	0.338
Marital status, *n* (%)			0.169
Married	1 (2.6)	2 (5.0)	
Never married	33 (86.8)	29 (72.5)	
Divorced	3 (7.9)	9 (22.5)	
Widowed	1 (2.6)	0 (0.0)	
Common‐law relationship	0 (0.0)	0 (0.0)	
Cohabitants, *n* (%)	35 (92.1)	28 (70.0)	0.007
History of physical illness, *n* (%)	6 (15.8)	8 (20.0)	0.911
History of psychiatric disorders, *n* (%)	25 (65.8)	32 (80.0)	0.245
First dextromethorphan overdose, *n* (%)	4 (10.5)	10 (25.0)	0.141
Source of dextromethorphan acquisition, *n* (%)			
Purchase from a retail store	27 (71.1)	31 (77.5)	0.607
Medication owned by a family member	1 (2.6)	2 (5.0)	0.518
Medication owned by a friend	1 (2.6)	0 (0.0)	0.487
Internet purchase	7 (18.4)	5 (12.5)	0.541
Others	0 (0.0)	3 (7.5)	0.241
Source of information on dextromethorphan overdose, *n* (%)			
SNS	14 (36.8)	13 (32.5)	0.641
Internet searches	19 (50.0)	10 (25.0)	0.020
Retail stores	3 (7.9)	9 (22.5)	0.117
Friends	7 (18.4)	5 (12.5)	0.536
Others	3 (7.9)	4 (10.0)	1
Purpose of overdose, *n* (%)			
Suicide/self‐harm	22 (57.9)	29 (72.5)	0.235
Escape from reality	16 (42.1)	11 (27.5)	0.235
Unable to stop	5 (13.2)	6 (15.0)	1
Wanting to feel better	3 (7.9)	3 (7.5)	1
Recreation/euphoria	5 (13.2)	1 (2.5)	0.104
Relaxation	3 (7.9)	1 (2.5)	0.352
Sleep	0 (0.0)	2 (5.0)	0.494
Seeking the intended effect	0 (0.0)	0 (0.0)	NA
None in particular	3 (8.1)	0 (0.0)	0.106
Others	8 (21.1)	4 (10.0)	0.219
Length of hospital stay, median [IQR]	2.0 [1.0–4.0]	2.0 [1.0–2.5]	0.623
Clinical outcome, *n* (%)			1
Death	0 (0.0)	0 (0.0)	
Residual sequelae	0 (0.0)	1 (2.6)	
Full recovery	37 (100.0)	38 (97.4)	
Follow‐up after discharge, *n* (%)			0.656
None	6 (15.8)	9 (23.1)	
Psychiatric department	27 (71.1)	26 (66.7)	
Non‐psychiatric department	1 (2.6)	2 (5.1)	
Both psychiatric and non‐psychiatric departments	4 (10.5)	2 (5.1)	
DAST‐20, median [IQR]	7.0 [5.0–10.0]	6.0 [5.0–8.0]	0.206
DAST‐20 Mild, *n* (%)	10 (29.4)	18 (48.6)	0.145

*Note*: The younger group was defined as patients aged 10–19 years, and the older group, as those aged ≥20 years. Multiple responses were allowed for the purpose of the overdose, source of dextromethorphan acquisition, and source of information on dextromethorphan overdose.

Abbreviations: IQR, interquartile range; SNS, social networking service.

## DISCUSSION

In this study, we examined the demographic characteristics and psychosocial backgrounds of patients who visited the ED for dextromethorphan overdose. Dextromethorphan overdose was mainly observed in young female patients. The following were commonly reported in the overall cohort: purchases from a retail store as the source of dextromethorphan acquisition, internet searches and SNS as sources of information on dextromethorphan overdose, and suicide/self‐harm as the purpose of overdose. In exploratory subgroup comparisons, for the younger age group, the proportion of patients with cohabitants was higher, and internet searches were more frequently reported as a source of information. However, these findings should be interpreted cautiously because no adjustment for multiple comparisons was performed.

This study showed that young women constituted a large proportion of patients who overdosed on dextromethorphan, which is consistent with the findings of recent Japanese studies on OTC medications. In Japan, epidemiological changes in OTC medication misuse have been observed before and after the COVID‐19 pandemic. Usami et al. analyzed data from the NPH Survey conducted before and after the COVID‐19 pandemic (2018 and 2022) and pointed out that the proportion of male individuals decreased from 60.0% to 44.3%, while the peak age distribution shifted downward from 30s–40s to 20s–30s.^6^ This trend appears to have persisted. In an ED‐based study by Kyan et al. in 2025, 79% of patients were female, with a median age of 22 years.^2^ Similarly, in another ED‐based study by Kohara et al. in the same year, women accounted for 61.5% of the cohort, with a mean age of 23.6 years.^3^ Although dextromethorphan misuse and overdose in the United States in the 2000s primarily involved younger individuals, no clear female predominance was observed. In a database study from a single poison control center in California, Banerji and Anderson reported that 85% of cases were concentrated among individuals aged 13–17 years, with a slight female predominance (46% male and 54% female).^11^ In addition, Bryner et al. analyzed multiple databases in the United States, including 1382 individuals, and showed that the median age was 16.0 years, and 59.9% were male.^12^

Although the sex distribution differed between recent reports from Japan and earlier reports from the United States, dextromethorphan misuse or overdose was more prevalent among adolescents and young adults in both settings. One neurobiological hypothesis is that the heightened reward sensitivity during adolescence contributes to this concentration. After overdose and systemic absorption, dextromethorphan is metabolized by cytochrome P450 2D6 (CYP2D6).^13^, ^14^ Dextrorphan antagonizes the *N*‐methyl‐d‐aspartate (NMDA) receptor,^15^ and this mechanism has been suggested to produce dissociative effects.^16^ In addition, because NMDA antagonism promotes dopamine release in the striatum,^17^ dextromethorphan may contribute to the development of dependence through a similar mechanism.^18^ Furthermore, it has been hypothesized that adolescence is characterized by an imbalanced activation of striatal dopaminergic circuits,^19^ and several studies have supported this concept using functional magnetic resonance imaging.^20^, ^21^, ^22^ However, this neurobiological explanation remains speculative in the context of this descriptive study.

Although no clear sex‐based difference has been identified in reports from the United States, several interrelated factors may contribute to the marked female predominance observed in the present study. Usami et al. suggested that women may have greater exposure to drugstores through the purchase of sanitary products and cosmetics and may be more familiar with nonprescription analgesics for menstrual pain, potentially lowering the psychological barrier to using OTC medications.^6^ In addition to these access‐related factors, psychiatric and behavioral factors may be relevant. In the present cohort, 82.1% of patients were female, 73.1% had a history of psychiatric disorders, and 65.4% reported suicide/self‐harm as a purpose of overdose. In addition, depressive disorders are more common among women in the general population,^23^ and prior literature suggests that women may be more likely to use substances in response to stress or negative affect.^24^ A Japanese emergency‐care study of high‐lethality suicide attempters also found that drug overdose was significantly more common among female than among male patients.^25^ Moreover, a recent nationwide survey of patients with drug‐related psychiatric disorders in Japan found that, compared with codeine misusers, dextromethorphan misusers included a higher proportion of female patients and had higher rates of mood disorders and histories of self‐harm or suicide attempts.^26^ Although these findings do not specifically demonstrate that female sex, psychiatric morbidity, and self‐harm were associated with one another within the dextromethorphan group, they suggest that these characteristics may cluster in the Japanese clinical population. Thus, the observed female predominance may partly reflect the intersection of psychiatric morbidity or psychological distress, self‐harm behavior, and the selection of readily accessible OTC medications, rather than any single factor alone.

The patterns of dextromethorphan overdose in Japan and the United States also appear to differ in purpose. In the present study, suicide/self‐harm was the most frequently reported purpose, consistent with previous Japanese ED studies of OTC drug overdose.^2^, ^3^ By contrast, recreational misuse of dextromethorphan became widespread among adolescents in the United States during the 2000s.^27^ In Japan, drug overdose has also been reported to be more common among female than male patients presenting to emergency care after suicide attempts.^25^ Thus, ED‐based studies may preferentially capture a clinical population in which both female patients and self‐poisoning are common, potentially contributing to the female predominance observed in the present study. However, because we did not examine sex‐stratified associations among psychiatric history, overdose purpose, and source of dextromethorphan acquisition, the extent to which these factors contributed to the observed sex distribution remains uncertain.

Moderate or higher DAST‐20 scores were observed in 60.6% of respondents in the present study, compared with 32.7% (17/52) in a previous Japanese study of patients with overdoses involving various OTC medications.^3^ In that study, DAST‐20 scores were also associated with histories of overdose and OTC drug abuse.^3^ In another multicenter Japanese study, dextromethorphan was more frequently involved in the habitual OTC drug overdose group than in the nonhabitual group (10/33 [30.3%] vs. 7/91 [7.7%], *p* = 0.001).^2^ However, because the study populations and outcome definitions differed, these findings should not be interpreted as evidence that dextromethorphan has greater dependence liability than other OTC ingredients. Rather, they highlight the importance of assessing repeated drug use, difficulty controlling drug use, and adverse consequences related to drug use in patients presenting with dextromethorphan overdose.

Among the patients enrolled in this study, internet searches were more frequently reported as a source of information by younger patients than by older patients. Other studies on OTC medication overdoses in Japan have shown that the internet occupies an important position as a source of information. In an ED‐based study conducted by Kohara et al., among 52 patients, 12 (23.0%) reported SNS and 22 (42.3%) reported internet searches as their source of information.^3^ A survey of Japanese high school students also indicated that adolescents who misuse OTC medications tend to search for information online more often and spend more time on SNS.^28^

The more frequent reporting of internet searches among younger patients may reflect age‐related differences in information‐seeking behavior. Previous qualitative research suggested that the accessibility and perceived anonymity of the internet may facilitate its use by young people seeking health‐ and medication‐related information.^29^ Among online sources, adolescents may intentionally seek health information through search engines, whereas they may encounter such information incidentally on SNS.^30^ In addition, adolescents may perceive health information on SNS as less trustworthy than information on websites and may use SNS primarily for communication and social connection rather than for purposeful health information seeking.^30^, ^31^ These differences may help explain why deliberate internet searches were more readily recognized and reported by younger patients as a source of information on dextromethorphan overdose, whereas information encountered incidentally on SNS may have been less likely to be identified as such. However, our study did not assess why patients selected particular information sources or how they evaluated their trustworthiness, and this interpretation remains speculative.

Among the patients enrolled in this study, internet searches and SNS were commonly reported as sources of information on dextromethorphan overdose. This pattern is consistent with previous reports from the United States describing online environments as sources of information related to dextromethorphan misuse.^32^

Historical experiences in the United States indicate that dextromethorphan misuse has been addressed through a combination of controlling sales and internet‐based preventive measures.^32^ In Japan, regulatory responses to the misuse of OTC medicines are also evolving, and additional measures—not only limited to internet‐based prevention^33^, ^34^ but also targeting the sale of products containing dextromethorphan and diphenhydramine—came into effect on May 1, 2026.^35^ Our findings, which included patients transported to emergency facilities between July 10, 2023, and December 31, 2025, are best interpreted as background data on the pattern of dextromethorphan overdose under the pre‐implementation regulatory environment.

This study had several limitations. First, because the study included only patients who presented to EDs in Japan after an overdose, patients who did not seek medical attention (e.g., because their symptoms and signs were mild) were not included; therefore, the study population may have been biased toward more severe cases. Accordingly, the findings may not be generalizable to individuals who experienced milder intoxication and did not seek medical attention or to those who were treated in other clinical settings. Second, although this was a multicenter prospective observational study, patients were not enrolled consecutively, and the screening logs of all potentially eligible patients were not maintained. Therefore, the number and characteristics of patients who were not approached or enrolled in the study are unknown. This may have introduced selection bias, and the included sample may not have fully represented all patients presenting with dextromethorphan overdose during the study period. This selection process may have affected the observed differences between the younger and older groups. Because consent from a parent or legal guardian was required for minors, younger patients who lived with or were accompanied by a parent or legal guardian may have been more likely to be enrolled. Consequently, the proportion of patients with cohabitants in the younger group may have been overestimated, potentially exaggerating the observed difference between the age groups. In addition, patients who were able or willing to provide detailed information about the ingested product and its information sources may have been more likely to be enrolled, potentially influencing the observed distribution of information sources. However, whether this selection process differed by age is unknown; therefore, its impact on the observed between‐group difference in internet searches cannot be determined. Third, the questionnaire recorded only whether patients identified SNS or internet searches as a source of information on dextromethorphan overdose. It did not assess the frequency or duration of internet use, specific websites or online platforms, the content or timing of the information encountered, or whether such information influenced the decision to overdose. Therefore, the observed finding represents an association between age groups and reporting internet searches as an information source and does not establish that internet exposure influenced overdose behavior. Fourth, the timing of DAST‐20 administration relative to the resolution of the acute effects of the overdose was not prespecified in the study protocol. Consequently, administration timing may have varied among patients and institutions, and residual toxic effects or differences in clinical condition at the time of questionnaire completion may have influenced the responses; however, this possibility could not be evaluated. Fifth, allowing multiple responses to determine the purpose of overdose made it possible to better reflect the complex psychological background of patients who overdosed; however, because we did not use structured instruments, such as the Mini International Neuropsychiatric Interview, to assess suicidality,^36^ the accuracy of this assessment was limited. In addition, although the presence or absence of a history of psychiatric disorders was recorded, specific psychiatric diagnoses were not collected. Therefore, the psychiatric backgrounds of the included patients could not be characterized in detail. Sixth, the subgroup analyses were exploratory, and no adjustment was made for multiple comparisons. Consequently, the statistically significant findings may reflect Type I error and should be interpreted with caution. Finally, this was a descriptive study; therefore, we could not control for potential confounding factors, and no causal inferences could be made. In particular, sex‐stratified associations among psychiatric history, overdose purpose, and source of dextromethorphan acquisition were not examined; thus, the mechanisms underlying the observed female predominance could not be directly evaluated.

Overall, among patients presenting to EDs in Japan for dextromethorphan overdose, internet searches were more frequently reported as a source of information in the younger group than in the older group. However, because this was a nonconsecutive observational study limited to patients who sought emergency care, the findings may not represent all individuals with dextromethorphan overdoses.

In conclusion, this study describes the demographic characteristics and psychosocial backgrounds of patients who presented to EDs in Japan after a dextromethorphan overdose. We found that patients who were predominantly young women, intent on suicide/self‐harm, who sourced internet‐based information, and obtained a moderate or greater DAST‐20 scores were commonly reported. These findings underscore the importance of comprehensive psychosocial assessment for individuals at risk of dextromethorphan overdose.

## AUTHOR CONTRIBUTIONS

Ryoko Kyan conceived and designed the study. Hironori Katsuki, Ryoko Kyan, Saeko Kohara, Hidetada Fukushima, Yuta Mizuno, Hidenobu Ochiai, Norio Otani, and Dai Taguchi acquired data. Hironori Katsuki and Michiko Takai analyzed and interpreted the data. Hironori Katsuki drafted the manuscript. Yoshito Kamijo, Ryoko Kyan, and Michiko Takai revised the manuscript critically for important intellectual content. All the authors approved the final version of the manuscript.

## CONFLICT OF INTEREST STATEMENT

The authors declare no conflicts of interest.

## ETHICS APPROVAL STATEMENT

The research related to human use complied with all relevant national regulations and institutional policies, in accordance with the tenets of the Helsinki Declaration, and was approved by the Institutional Review Board of the Ethics Committee of Saitama Medical University Hospital (2023‐027).

## PATIENT CONSENT STATEMENT

All participants provided consent during the study period.

## CLINICAL TRIAL REGISTRATION

N/A.

## Data Availability

Raw data were generated at Saitama Medical University Hospital. Data supporting the findings of this study are available from the corresponding author, H.K., upon request.
